# Atypical telencephalic reflux pattern in a cavernous sinus dural AV fistula related to an anatomical variation of the basal vein of Rosenthal

**DOI:** 10.1177/15910199241260758

**Published:** 2024-06-07

**Authors:** Shigeta Miyake, Tze P Kee, Andrew Falzon, Hugo Andrade, Timo Krings

**Affiliations:** 1 Department of Neurosurgery, 13155 Yokohama City University School of Medicine, Yokohama, Kanagawa, Japan; 2 Division of Neuroradiology, Department of Medical Imaging, Joint Department of Medical Imaging, University Health Network and 563025 Toronto Western Hospital, University of Toronto, ON, Canada; 3 Department of Neuroradiology, 54738 National Neuroscience Institute, Singapore; 4 Division of Neurosurgery, Sprott Department of Surgery, 26625 Toronto Western Hospital, University of Toronto, ON, Canada

**Keywords:** Dural arteriovenous fistula, cavernous sinus, telencephalon, basal vein of Rosenthal, anatomy, embryology

## Abstract

Cavernous sinus dural arteriovenous fistula can cause cerebral edema and hemorrhage due to cortical venous reflux and congestion. Understanding complex venous reflux and drainage routes is crucial for treatment planning. Here, we present a case of a cavernous sinus dural arteriovenous fistula with cortical venous reflux via two separate terminations of the telencephalic veins caused by an aplastic basal vein of Rosenthal. The patient presented with diplopia and eye redness and was diagnosed with a Cognard type IIa + b cavernous sinus dural arteriovenous fistula. The shunt was supplied by the dural branches of the internal and external carotid arteries. Multiple shunt points involving the intercavernous sinus and the medial aspect of the left cavernous sinus were identified, with drainage into the supraorbital and intracranial veins, including two separate terminations of the telencephalic veins, one leading to the laterocavernous sinus via the superficial middle cerebral vein and the other to the cavernous sinus via the uncal vein, resulting in basal ganglia venous congestion in the absence of the basal vein of Rosenthal. During transvenous embolization, the intracranial veins, cavernous sinus, and intercavernous sinus were obliterated using a double-catheter technique with a combination of coils and liquid embolics. Telencephalic venous variations can lead to cavernous sinus drainage into the basal ganglia and orbitofrontal brain. This unique drainage pattern underscores the importance of recognizing anatomical variations when managing cavernous sinus dural arteriovenous fistula.

## Introduction

The cavernous sinus (CS) is a common site for dural arteriovenous fistulas,^1^ and can exhibit a multidirectional drainage pattern.^2^ Typical drainage routes include anterior drainage via the superior and inferior ophthalmic veins, inferior drainage via the inferior petrosal sinus (IPS) and pterygoid plexus, posterior drainage through the superior petrosal sinus, medial drainage via the intercavernous sinus (ICS), lateral drainage through the superficial middle cerebral vein (SMCV), and deep drainage through the prepontine bridging vein (PPBV) and uncal vein (UV) to the basal vein of Rosenthal (BVR).^3^ The telencephalic venous group, which forms the first segment of the BVR, is particularly recognized as a potential cortical venous reflux (CVR) of CS dural arteriovenous fistula (CSDAVF).^2,4,5^

Although cerebral edema associated with CVR is rare in CSDAVF,^2,5^ incomplete obliteration of deep and cortical venous drainage, causing high-pressure rerouting into the deep or cortical venous system, has been reported to lead to fatal cerebral edema and hemorrhage.^6^ Herein, we present a rare case of CSDAVF with extensive venous reflux to the left cerebral hemisphere caused by anatomical variations in the BVR. Identifying this unusual termination pattern in the veins is a crucial first step in treatment planning and subsequent successful embolization.

## Case presentation

### Clinical presentation and imaging

**Figure 1.**
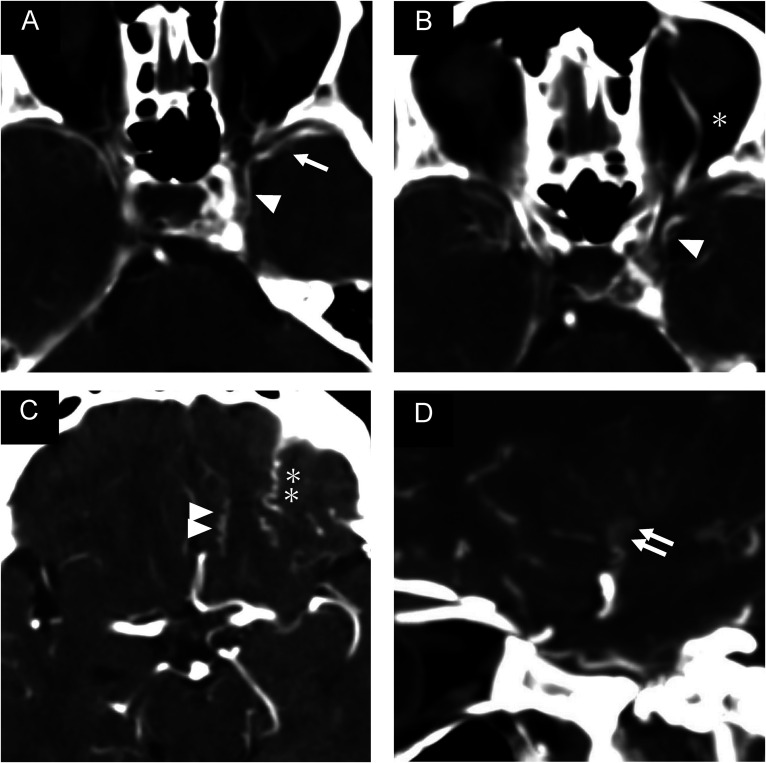
Pre-procedural head computed tomography angiogram showing early contrast opacification of the left cavernous sinus and left superficial middle cerebral vein (single arrow) connected to the laterocavernous sinus (single arrowhead) (A). Multidirectional drainage routes including the superior ophthalmic vein (B, single asterisk), olfactory vein (C, double arrowhead), orbitofrontal vein (C, double asterisk), and inferior striate vein (D, double arrow) can be observed.

**Figure 2.**
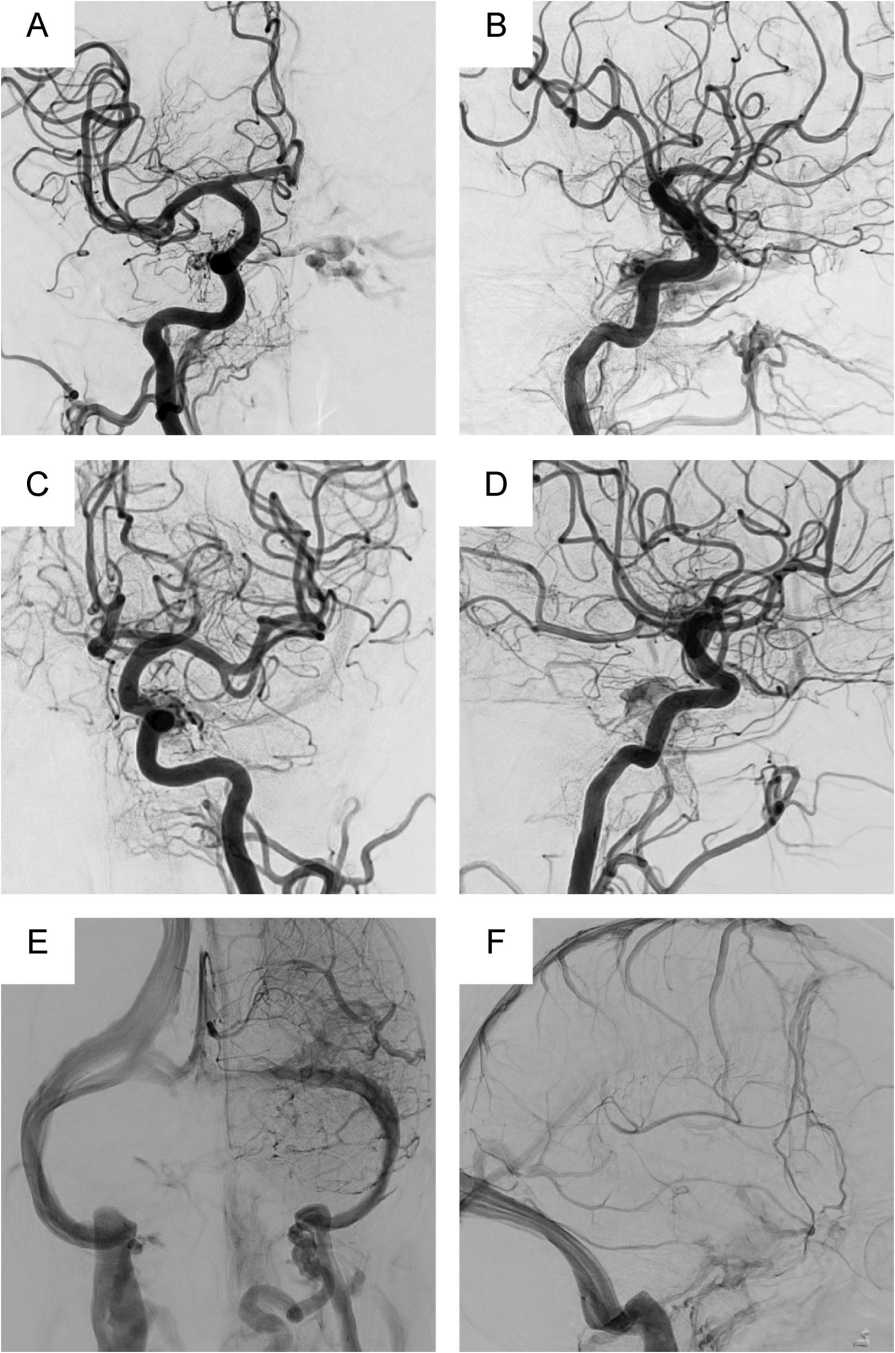
Antero-posterior and lateral views of the right common carotid angiography (A and B: Arterial phase), left common carotid angiography (C and D: Arterial phase, E and F: Venous phase) are shown. The right internal carotid artery (meningo-hypophyseal trunk), left internal carotid artery (inferolateral trunk), and bilateral external carotid artery (petrosal branch of the middle cerebral artery, artery of foramen rotundum, and ascending pharyngeal artery) can be observed to feed into this shunt with a diffuse shunt point extending from the intercavernous sinus to the medial aspect of the cavernous sinus. There was no reflux to the inferior petrosal sinus and basal vein of Rosenthal (BVR). The BVR could not be observed even in the venous phase, and the BVR was thus considered hypoplastic or aplastic.

A 73-year-old female presented to our hospital with a 1-month history of diplopia. Computed tomography (CT) of the head revealed no evidence of intracranial hemorrhage, cerebral edema, or mass effects. CT angiography further revealed early contrast opacification of the left CS with a dilated left superior ophthalmic vein (SOV) and left SMCV (Figure 1(A) and (B)). Dilated olfactory vein (OlfV), orbitofrontal vein (OFV), inferior striate vein (ISV), and deep middle cerebral veins (DMCVs) were observed, indicating a high-grade fistula (Figure 1(C) and (D)). Digital subtraction angiography (DSA) revealed a CSDAVF supplied by the meningo-hypophyseal trunk of the right internal carotid artery (ICA), the inferolateral trunk arising from the left ICA, the ascending pharyngeal artery, the petrosal branch of the middle meningeal artery, and the artery of the foramen rotundum of the bilateral external carotid artery (Figure 2(A) to (D)). Multiple shunt points involving the ICS and medial aspect of the left CS, with drainage into the SOV and intracranial veins, including the SMCV, UV, and PPBV, were identified. The left IPS was occluded, while the right IPS was only partially occluded at the proximal segment and was patent distally. Interestingly, BVR was not detected in the entire venous phase (Figure 2(E) and (F)), consistent with hypoplasia. Transvenous embolization (TVE) was performed in the view of the high-grade nature of the fistula (Cognard type IIa + b CSDAVF) and the symptomatic presentation.

### Treatment

**Figure 3.**
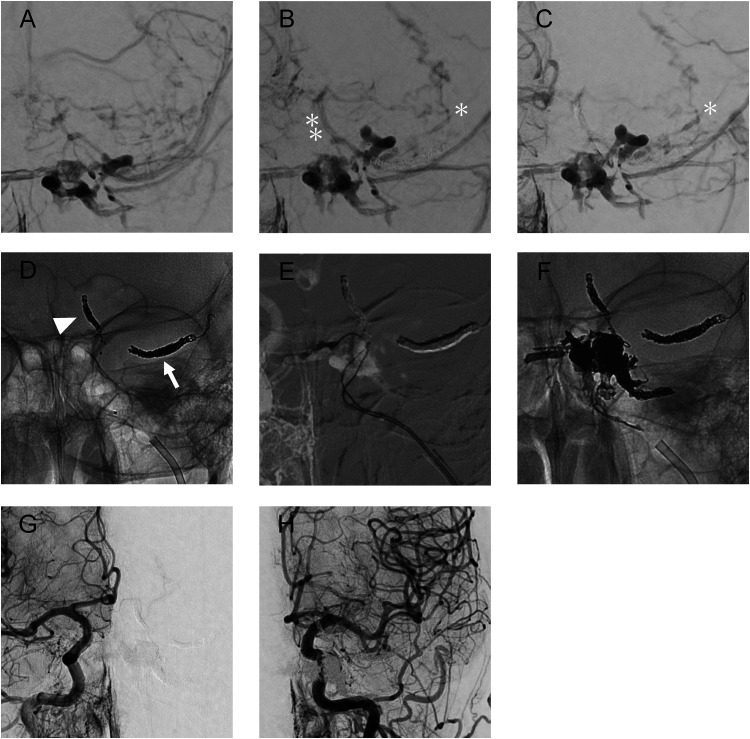
The patient's stepwise treatment course is shown, with the results of digital subtraction angiography and digital angiography. Antero-posterior images of the right external carotid angiography (A: pre-procedure, B: after embolization of the SMCV, and C: after embolization of the UV) showing the progression of gradual embolization of the SMCV and UV (D, arrow and arrowhead, respectively). After embolization of the SMCV (B), as obvious reflux to the orbitofrontal vein (OFV, single asterisk) and UV (double asterisk) could be observed. Following embolization of the SMCV and UV, reflux of the OFV originating from the proximal part of the SMCV can be seen. The double catheter technique (E) was used for complete obliteration. Initially, the superior ophthalmic vein, sphenoparietal sinus, and CS were tightly packed with coils, and the shunt was completely blocked with ONYX from the catheter placed in the medial part of the CS (F). Finally, the complete obliteration was confirmed with bilateral common carotid angiography (G and H). UV: uncal vein; OFV: orbitofrontal vein; SMCV: superior middle cerebral vein; CS: cavernous sinus.

TVE was performed by carefully tunneling the occluded left IPS using a microcatheter microwire assembly (Figure 3). To effectively occlude all fistulous points along the ICS and left CS, as well as the refluxed venous channels, including the SMCV, UV, PPBV, and SOV, a double catheter embolization technique was employed to sequentially block off all venous exits of the CS. First, the SMCV was targeted and embolized using coils. Next, the UV leading to the confluence of the DMCV and anterior cerebral vein (ACV) was embolized with coils. Another microcatheter, the microwire assembly, was navigated through the left IPS alongside the first microcatheter to the left CS. Subsequently, the ICS, SOV, and sphenoparietal sinus were sequentially embolized, leaving one catheter in the medial part of the CS. Finally, the injection of a small amount of liquid embolic (Onyx) to occlude the remaining fistula in the ICS and left CS was performed. The patient recovered well after the procedure, with no new neurological deficits or worsening visual symptoms.

### Variant termination of the telencephalic veins

**Figure 4.**
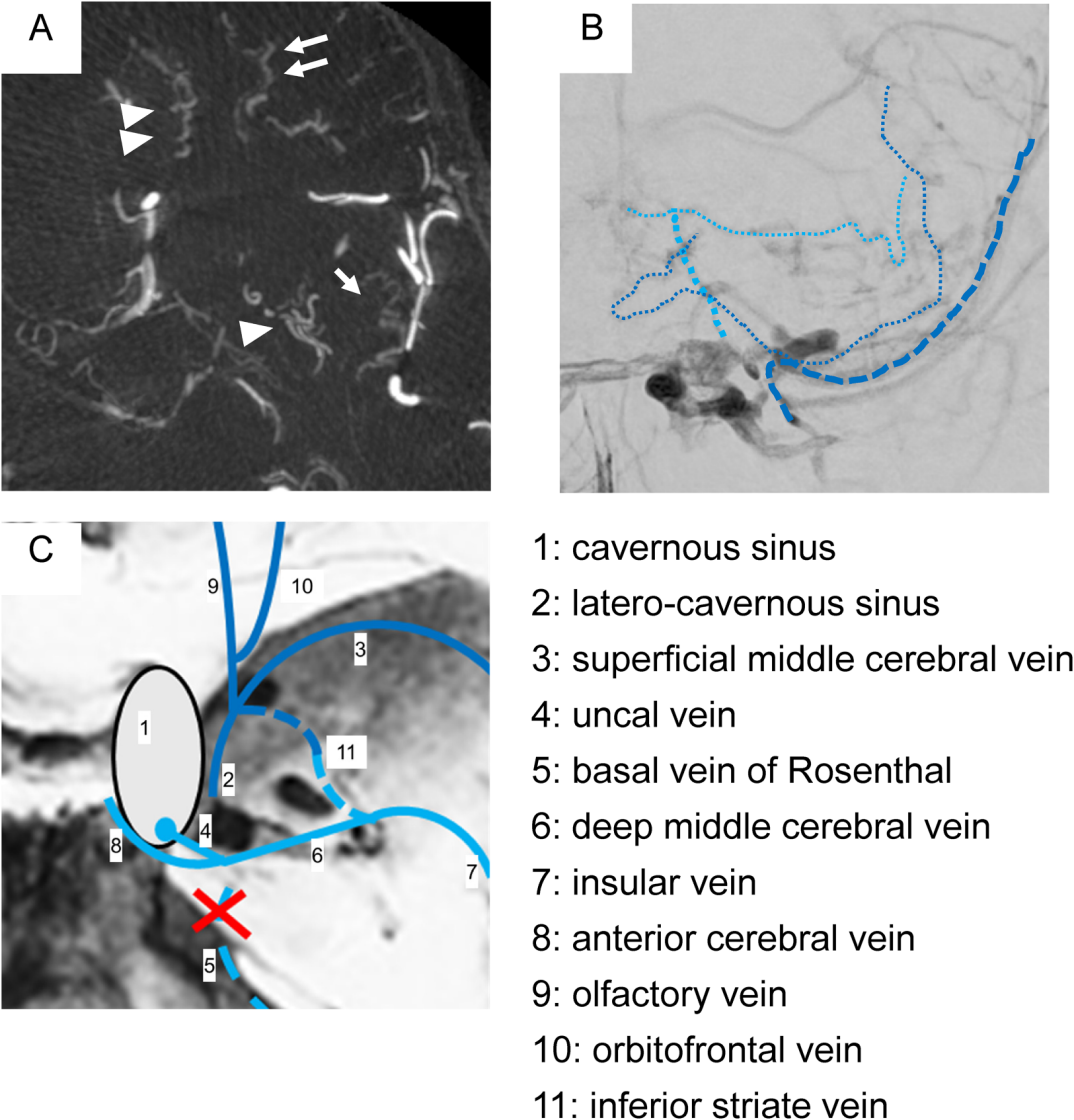
Reflux and termination of telencephalic veins are shown in the axial image of the maximum intensity projection (A), anteroposterior image of right external carotid angiography (B), and an illustration (C). Obvious dilated veins (inferior striate vein (single arrowhead), insula vein (single arrow), olfactory vein (double arrowhead), and orbitofrontal vein (double arrow)) are shown (A). Termination to the cavernous sinus via the uncal vein (thin blue dotted line) and to the superficial middle cerebral vein (dark blue dotted line) is evident in the pre-procedural imaging. The venous terminations, in this case are illustrated (1: cavernous sinus, 2: latero-cavernous sinus, 3: superficial middle cerebral vein, 4: uncal vein, 5: basal vein of Rosenthal, 6: deep middle cerebral vein, 7: insular vein, 8: anterior cerebral vein, 9: olfactory vein, 10: orbitofrontal vein, 11: inferior striate vein).

Variant termination of the telencephalic veins was demonstrated by DSA. The BVR was hypoplastic, which resulted in a variant organization of the telencephalic venous drainage. Preprocedural imaging revealed dilatation of the SMCV, UV, DMCV, ACV, OlfV, OFV, and ISV from the venous reflux (Figures 3(A), 4(A) and (B)). Stepwise coil embolization of the refluxed venous channels revealed distinct groupings of the telencephalic veins in the absence of a well-developed BVR. The reflux into the SMCV was eliminated by coiling the SMCV (Figure 3(B)). The reflux to the ACV and DMCV was markedly reduced after coiling the UV (Figure 3(C)). As the OlfV and OFV drained into the SMCV near the laterocavernous sinus (LCS), venous reflux into the OlfV and OFV was eliminated following complete embolization of the CS with coils and liquid embolics (Figure 3(G) and (H)).

## Discussion

In the present case, endovascular cure was achieved by sequentially occluding the individual channels of CVR from the CSDAVF before occluding the shunting zones along the ICS and left CS. The variant venous drainage pattern in the absence of a normal anatomical disposition of the BVR, leading to distinct groups of telencephalic venous terminations to the LCS via the SMCV and to the CS via the UV, was of particular interest.

In general, reflux to the UV can result in reflux to the basal ganglia via the ISV, the insular cortex via the DMCV, and the frontal lobe via the OlfV and OFV.^7^ The patency of drainage routes, such as the BVR, is important in the presence of venous reflux as it determines the potential impact of reflux.^8^ There have been several reports of an association between BVR segment connectivity and BVR thrombosis, with intracranial hemorrhage and cerebral edema.^9–11^ Given that disconnection of the first segment of the BVR is reported to occur in 10%–30%^9,10^ the risk of symptomatic CVR with CSDAVF should not be underestimated.^2^

Venous reflux involving the basal ganglia region is rare.^2^ Reports of reflux/cerebral edema/intracerebral hemorrhage to the basal ganglia include one case report of reflux to the ISV in a direct carotid cavernous fistula,^12^ and one case of reflux to the UV in a direct carotid cavernous fistula without the posterior segment of the BVR.^13^ Additionally, Ide et al. reviewed 26 cases of CSDAVF and reported that direct reflux into the UV resulted in putaminal hemorrhage with perifocal edema in one case.^14^ Similarly, Miyamoto et al. reviewed 54 cases of CSDAVF, involving cerebral edema and hemorrhage in 6 cases, of which two showed involvement of the deep Sylvian vein.^8^ Thus, symptomatic reflux to the basal ganglia occurs in approximately 4% of patients with CSDAVF. Anatomically, the connection from the CS to the UV was reported to be present in 50% of cases,^14^ among which reflux to the UV was recognized to account for 15% of CVR.^5^ In addition to the main drainage route of the BVR, the drainage route from the ISV to the intraventricular veins via the superior striate vein^15,16^ may contribute to lowering cerebral edema of the basal ganglia.

Considering the venous reflux to the telencephalic venous group in CSDAVF, variations in the termination patterns of the telencephalic venous group are important.^14^ In the present case, the cortical veins (OlfV and OFV) terminated directly proximal to the SMCV, whereas the deep veins (DMCV and ACV) terminated at the CS via UV (Figure 4(B) and (C)). Regarding the termination patterns of telencephalic venous groups, it has been reported that UV and BVR are complementary,^8^ and that termination of the SMCV varies depending on the degree of CS capture.^17^ Furthermore, the diversity of UV terminations is believed to be caused by the development of the deep telencephalic vein (DTV), which is the origin of UV and DMCV, and the superficial telencephalic vein (STV), which is the origin of SMCV.^14^ The formation of the CS as a ventral drainage pathway, caused by the elongation and extinction of the primitive tentorial sinus (PTS) due to brain expansion, results in diverse UV terminations, depending on whether UV is attributed to the CS or the SMCV and the degree of CS capture by the SMCV.^18^ Although the drainage patterns of the telencephalic venous groups have been studied, the formation of networks within these groups has not yet been reported.

Anatomically, the OlfV and OFV are located on the inferior surface of the frontal lobe. The ACV runs laterally along the superior-lateral border of the optic chiasm and optic tract, originating from the insula, crosses medially through the anterior perforate substance, and merges with the ACV.^7^ The ISV is derived from the anterior perforated substance and primarily merges with the DMCV. Although the DMCV primarily connects with the BVR, it sometimes flows into the SMCV. Similarly, the OlfV and OFV often terminate in the DMCV via the ACV; however, there have been reports of these vessels merging and terminating in the SMCV. Additionally, ISV is reported to derive into the OlfV as well as into the DMCV.^19^ This means that the anteriorly located OlfV and OFV may terminate in the SMCV, while the ISV may terminate both anteriorly and posteriorly (Figure 4(C)). This termination pattern of the telencephalic veins may be embryologically attributable to the affiliation of the STV and DTV. As such, there is a potential for anastomosis in the telencephalic veins of the inferior surface of the frontal lobe, particularly if the BVR does not form contemporaneously with regression of the PTS. The drainage route of the frontal lobe may be the SMCV originating from the STV or the CS via the UV originating from the DTV. Alternatively, both may remain, as in the present case. Variability in the BVR may contribute not only to the risk of symptomatic CVR^5,8^ in CSDAVF, but also to the variability of telencephalic vein terminations.

In conclusion, this experience shows that the presence of reflux to the basal ganglia and separate termination of the telencephalic venous group in CSDAVF highlights the anatomical complexity and variability of venous drainage patterns in CSDAVF. The existence of this variation is not only clinically significant, but also important in understanding the development of the telencephalic venous group in the formation of the BVR and CS. Recognizing and adequately addressing these variations is crucial to ensure complete obliteration and successful management of CSDAVF.
